# Analysis of Mutational Profile of Hypopharyngeal and Laryngeal Head and Neck Squamous Cell Carcinomas Identifies *KMT2C* as a Potential Tumor Suppressor

**DOI:** 10.3389/fonc.2022.768954

**Published:** 2022-05-19

**Authors:** Marcin M. Machnicki, Anna Rzepakowska, Joanna I. Janowska, Monika Pepek, Alicja Krop, Katarzyna Pruszczyk, Piotr Stawinski, Malgorzata Rydzanicz, Jakub Grzybowski, Barbara Gornicka, Maciej Wnuk, Rafal Ploski, Ewa Osuch-Wojcikiewicz, Tomasz Stoklosa

**Affiliations:** ^1^ Department of Tumor Biology and Genetics, Medical University of Warsaw, Warsaw, Poland; ^2^ Department of Otorhinolaryngology, Head and Neck Surgery, Medical University of Warsaw, Warsaw, Poland; ^3^ Department of Immunology, Medical University of Warsaw, Warsaw, Poland; ^4^ Department of Medical Genetics, Medical University of Warsaw, Warsaw, Poland; ^5^ Department of Pathology, Medical University of Warsaw, Warsaw, Poland; ^6^ Department of Biology, University of Rzeszow, Rzeszow, Poland

**Keywords:** laryngeal cancer, Kmt2c, MLL3, mutational landscape, head and neck squamous cell carcinoma (HNSCC), hypopharyngeal cancer (HPC), next-generation sequencing (NGS)

## Abstract

Hypopharyngeal cancer is a poorly characterized type of head and neck squamous cell carcinoma (HNSCC) with bleak prognosis and only few studies focusing specifically on the genomic profile of this type of cancer. We performed molecular profiling of 48 HPV (Human Papilloma Virus)-negative tumor samples including 23 originating from the hypopharynx and 25 from the larynx using a targeted next-generation sequencing approach. Among genes previously described as significantly mutated, *TP53, FAT1, NOTCH1, KMT2C*, and *CDKN2A* were found to be most frequently mutated. We also found that more than three-quarters of our patients harbored candidate actionable or prognostic alterations in genes belonging to RTK/ERK/PI3K, cell-cycle, and DNA-damage repair pathways. Using previously published data we compared 67 hypopharyngeal cancers to 595 HNSCC from other sites and found no prominent differences in mutational frequency except for *CASP8* and *HRAS* genes. Since we observed relatively frequent mutations of *KTM2C (MLL3)* in our dataset, we analyzed their role, *in vitro*, by generating a *KMT2C*-mutant hypopharyngeal cancer cell line FaDu with CRISPR-Cas9. We demonstrated that *KMT2C* loss-of-function mutations resulted in increased colony formation and proliferation, in concordance with previously published results. In summary, our results show that the mutational profile of hypopharyngeal cancers might be similar to the one observed for other head and neck cancers with respect to minor differences and includes multiple candidate actionable and prognostic genetic alterations. We also demonstrated, for the first time, that the *KMT2C* gene may play a role of tumor suppressor in HNSCC, which opens new possibilities in the search for new targeted treatment approaches.

## Introduction

Despite several new treatment modalities tested in the last decade, head and neck squamous cell carcinoma (HNSCC) remains one of the five most common human cancers with significant morbidity and mortality worldwide. Among HNSCC, hypopharyngeal cancer was diagnosed in more than 80,000 individuals in 2018 (0.4% of all HNSCC) and was responsible for almost 35,000 deaths (0.4% of all cancer-related deaths) (1). Smoking, human papilloma virus (HPV) infection, and alcohol consumption are among major risk factors for this cancer. Although hypopharyngeal cancer is rare and accounts for 2–14% of malignancies in the HNSCC group (2–4), the clinical prognosis is very poor, despite aggressive multidisciplinary treatment protocols. The 5-year overall and disease-specific survival rates remain at 30–35% in contrast to the laryngeal cancer (5–7). Unfavorable prognosis results from a large percentage (60–85%) of newly diagnosed patients with hypopharyngeal cancer presenting in advanced stages of the disease (III–IV) (6–8). Asymptomatic progression in early disease stages, the tendency of submucosal spread, and the high number of lymphatic vessels in hypopharyngeal mucosa contribute significantly to the higher tumor stage (tumor-node-metastasis, TNM) (4, 9). Currently, the most important prognostic indicators of hypopharyngeal cancer, similarly to other head and neck cancers, are patient age and tumor stage. Consistently with other head and neck cancers, squamous cell carcinoma is the most common histological type of hypopharyngeal carcinoma (10). In contrast to other types of head and neck cancers originating from larynx, oral cavity, and oropharynx, the incidence rates of local recurrence, nodular metastasis, and second primary tumors are significantly higher for hypopharyngeal cancer (4, 9, 11). The introduction of new treatment protocols in the 1990s from primary surgical resection to definitive radiation therapy combined with induction or concurrent chemotherapy, did not appear effective in improving survival rates in hypopharyngeal cancer (12). Moreover, in advanced cancers involving both the larynx and hypopharynx, it may not be possible to define the primary site. Unfortunately, still no individualized therapeutic options can be recommended for hypopharyngeal cancer patients. Therefore, comprehensive analysis of genetic alterations in clinically and histopathologically confirmed hypopharyngeal and laryngeal cancers may help to better understand significant differences in the molecular pathogenesis of both cancers.

Currently, the identification of the underlying molecular mechanisms involved in hypopharyngeal cancer with analysis of differentially expressed genes, key functional pathways, and molecular biomarkers gives the most promising opportunity to improve the efficacy of diagnostic and therapeutic strategies among patients. Although HNSCC genetics have been widely explored by large consortia such as TCGA, these studies have significant underrepresentation of hypopharyngeal cancer (13, 14). It has been recently updated and partially supplemented by Vossen et al., who performed a comprehensive study focused on oropharyngeal cancer compared with the larynx and hypopharyngeal tumors (15). However, the genetic landscape of this type of tumor is still only partially understood. Importantly, there is still no translation of the described genetic aberrations into better stratification of patients or what is most desired: into clinical benefit.

Therefore, we performed a comprehensive analysis of the molecular landscape of hypopharyngeal cancer and laryngeal cancer to analyze the mutation frequency in major genes and functional pathways associated with oncogenesis as well as to identify clinically relevant and recurrent genetic aberrations. Additionally, we compared data from our hypopharyngeal tumors with previously published results to better characterize mutational profile of this cancer. From the notable genetic aberrations identified in our patients, we have selected the *KMT2C* gene as a potential tumor suppressor inactivated in a significant proportion of patients. The role of *KMT2C* inactivation is not characterized in this cancer in contrast to other top-mutated genes such as *TP53* or *NOTCH1*. We tested the potential mechanistic role of *KMT2C* gene aberrations in a FaDu hypopharyngeal cell line model using CRISPR-Cas9-targeted gene inactivation followed by functional assays.

## Materials and Methods

### Patients and Pathologic Classification

A total of 48 samples were obtained from HNSCC patients and included in the analysis (**Table 1**) and clinical and pathological data tumors were rigorously classified as hypopharyngeal or laryngeal. Blood samples were collected from 13/48 patients prior to chemo-/radiotherapy or surgical intervention and those blood (normal)-tumor pairs were subject to exome sequencing. All of the diagnostic protocols were reassessed in order to standardize them with the 8^th^ Edition of the American Joint Committee on Cancer Staging Manual (16). In dubious cases, histologic slides were re-examined. As pathologic TNM classification is applicable to postoperative material, we were not able to perform staging in 6 cases in which only small biopsies were available. Histological types were consistent with the 4th edition of the WHO classification of Head and Neck Tumors (17), however in 5 cases a paucity of material did not allow to distinguish between keratinizing and nonkeratinizing subtypes of conventional squamous cell carcinomas (SCC). In few cases material was not originally properly designated and the samples were no longer available (those cases are marked as not determined in the **Table 1**).

**Table 1:** Patients’ characteristics.

Parameter	Value
Number of patients	48
Age [median (range)] [years]	60 (43 – 79)
Sex [n, %]	
Male	45 (94)
Female	3 (6)
Localization (primary site) [n,%]	
Hypopharynx	23 (48)
Larynx	25 (52)
Histological type [n,%] SCC:	
1. Conventional	
Keratinizing	32 (67)
Nonkeratinizing	8 (17)
2. Basaloid	3 (6)
3. Not determined	5 (10)
Pathologic Stage (pTNM) [n,%]	
pT1N1	1 (2)
pT2 (N0/1/2/3/x)	8 (17)
pT3 (N1/2/x)	12 (25)
pT4 (N1/2/3/x)	21 (44)
Not determined	6 (12)

### Nucleic Acid Isolation

DNA was isolated using either Gentra Puregene kit (Qiagen) from fresh or snap-frozen tumor tissues and cell line pellets or with DNA Blood Mini Kit (Qiagen) from patients’ blood samples. DNA was dissolved in Buffer AE (Qiagen) and stored at +4°C. A single tumor sample was isolated from formalin-fixed paraffin-embedded tissue using E.Z.N.A. FFPE DNA Kit (Omega Bio-tek).

### Human Papilloma Virus Testing

HPV infection status was assessed using PCR with MY11/09 primers as described previously, with modifications (18). A 20µl PCR mixture contained the following components: 1U of HotStartTaq Plus polymerase (Qiagen), 2µl 10x CoralLoad Buffer, 2×0.4µl MY09/MY11 primers (10µM), 0.4µl dNTPs (10mM), 0.8µl DNA (200ng) and 15.88 µl water. Cycling conditions were set as follows: 35 cycles (60 s at 94°C, 60 s at 55°C, 60 s at 72°C) with initial denaturation for 5 min at 95°C and final extension for 7 min at 72°C.

### TERT Promoter Sequencing

DNA sequences of *TERT* promoter (*pTERT*) were obtained in two reactions. The PCR reaction was performed with two primers: forward 5’- CACCCGTCCTGCCCCTTCACCTT and reverse 5’- GGCTTCCCACGTGCGCAGCAGGA (10 µM) (19) and the KAPA HiFi HotStart Ready Mix (KAPA Biosystems). The reaction mixture (20 µl) contained the following components: water - 7.8 µl, HotStart Mix - 10 µl, primers (forward and reverse) - 2×0.6 µl (10µM) and DNA - 1 µl (approx. 100-300ng). PCR was performed for 32 cycles (20 s at 95°C, 30 s at 72°C) with initial denaturation for 3 min at 95°C and final extension for 1 min at 72°C. The reaction resulted in formation of a 147 bp product. Subsequently, amplicon was purified using VAHTS DNA Clean Beads (Vazyme) (with a 1:1.8 DNA to beads ratio) and labeled using BigDye Terminator v3.1 Cycle Sequencing kit (Thermo Fischer Scientific, USA) and primers used in the first reaction. The reaction mixture (10 µl) contained the following components: BigDye - 0.4 µl, Sequencing Buffer - 3.6 µl, water - 2 µl, primer (forward or reverse, 1 µM) - 2 µl and 2 µl of the purified amplicon. PCR was performed in a thermocycler for 55 cycles (10 s at 95°C, 15 s at 50°C, 90 s at 60°C) with an initial denaturation for 5 min at 95°C and a final extension for 5 min at 60°C. All PCR amplicons were analyzed on 1-2% agarose gels stained with ethidium bromide (Sigma-Aldrich, USA).

Finally, 10 µl of labeled product was purified using beads by incubating the product for 5 min with 10 µl beads and with 42 µl of 90% ethanol at room temperature, two washes with 90% ethanol for 30 seconds on a magnet and elution with 10 µl of water. The product was then processed for sequencing on a Genetic Analyzer 3500 (Applied Biosystems, Thermo Fischer, USA). Chromatograms were analyzed using FinchTV software.

### Next-Generation Sequencing (NGS)

The whole process of library construction and enrichment was carried out using SeqCap EZ chemistry (Roche NimbleGen, USA), according to the SeqCap EZ Library SR User’s Guide v.4.2, with minor modifications, briefly described below. DNA from patient samples and FaDu cell lines was converted into DNA fragment libraries using KAPA Lib Prep kit (Kapa Biosystems, USA). 100–1000ng of DNA was sheared on Covaris M220 for 225s (175s for partially fragmented FFPE DNA) and used as an input for library construction, followed by end-repair, adenylation, and adapter ligation steps. The resulting libraries were then subject to dual-sided SPRI size-selection method and PCR amplification for 3-7 cycles, depending on DNA input. Subsequently, libraries were mixed into 8- to 24-plex pools, hybridized to SeqCap EZ capture probes, and reamplified for 9–10 PCR cycles.

For custom sequence capture SeqCap EZ custom probe designs targeting 7 Mb and 10Mb were used and for exome sequencing SeqCapEZ Exome v2 or MedExome (Roche NimbleGen) probe sets were employed. Additionally, TruSeq library preparation and exome enrichment kits (Illumina, USA) were used according to user guides for sequencing of three normal-tumor sample pairs. Regions not overlapping between custom/exome panels were excluded from analyses when needed. A list of probe designs used for individual samples is provided in **Supplementary Data 1.2**.

DNA and DNA library concentrations were measured on NanoDrop (Thermo Fisher Scientific) and Qubit fluorometer using dsDNA High Sensitivity kit (Thermo Fisher Scientific). Quality of DNA and DNA library fragment size ranges were assessed on 0.7–2% agarose gels or 2100 Bioanalyser instrument (Agilent Technologies, USA) when needed.

All libraries were sequenced on Illumina HiSeq 1500 instrument using 2 x 100 bp reads. Tumor samples analyzed with custom panels were sequenced to reach mean coverages in a range of 49.2–234x (median 154.93x) with % bp @ 20x in a range of 77.7–97.2% (median 95.6%) while tumor exome samples were sequenced to reach mean coverages in a range of 31.5– 145.2x (median 112.3x) and % bp @ 20x in a range of 71.4–99.2% (median 92.2%). Blood samples were sequenced to reach mean coverages in a range of 52.6–166.9x (median 93.4x) and % bp @ 20x in a range of 80.1–98.5% (median 89.8%).

### NGS Data Acquisition and Analysis

Raw sequencing data was processed according to Broad Institute recommendations. Variant discovery included the following steps: quality control of raw fastq, adapter trimming and low-quality reads removal using Trimmomatic (20), read mapping to hg19 using BWA-MEM (21), duplication removal, local realignment and quality recalibration using GATK and Picard and variant calling using UnifiedGenotyper, and HaplotypeCaller. Exome data was additionally analyzed using Mutect-2 to identify somatic single nucleotide variants (SNV) and insertions-deletions (indels) through direct comparison of germline and tumor samples (22).

Variants were filtered using public (NHLBI ESP (23), gnomAD (24) and internal databases in order to remove common genetic variation. CADD (25), PolyPhen2 (26), CHASM (27), SIFT (28), FATHMM (29) and Mutation Taster (30) predictions were used to identify possible protein-damaging missense alterations. All variants were also manually curated to avoid including sequencing artifacts and ClinVar (31), Varsome (32) and COSMIC (33) databases were also used to aid final variant classification. Complete lists of classified variants are available in **Supplementary Data 3**.

Copy-number calling was performed by sequencing coverage analysis using CNVkit v0.9.5 (34). CNVkit was run with default settings except for 400bp bin size limit. Groups of normal samples were used to create reference coverage models across predefined targets for each custom/exome capture. Two samples were recentered due to clear deviation of basal copy number. Additionally, a GISTIC 2.0.23 (35) analysis was performed on segmented CNV data acquired for genomic regions targeted by all custom captures (**Supplementary Data 1.3**). GISTIC was run with default parameters except for 5000bp pseudo-markers spacing setting. TCGA CNV data was reanalyzed with identical GISTIC setting. RAW CNV segmentation data for patients and cell lines is available in **Supplementary Data 3.2, 3.5**.

Heterozygosity alterations were analyzed across covered regions by plotting a chart of the observed absolute variant allele frequency (VAF) deviations from 0.5 (heterozygous state) for all variants called by HaplotypeCaller, with allele frequencies in range (0.0001, 0.95) in gnomAD and coverage larger than 30x. Normal-tumor pairs were additionally analyzed in a similar manner that also included calculation of VAF shifts between normal and tumor samples. This data was also segmented using CNVkit built-in cbs method after exclusion of homozygous variants to aid in identification of regions with allelic imbalance. Ambiguous results with a VAF deviation lower than 0.15 across segment were considered not altered.

External mutational data for HPV-negative (HPVneg) head and neck cancers was downloaded *via* cBioPortal (36, 37) for TCGA PanCancer Atlas (38), Agrawal et al. (13) and Stransky et al. (14) projects or from the journal’s site for Vossen et al. (15) project (referred also as “NKI dataset”). Datasets from those projects combined with a current dataset referred to as “Medical University of Warsaw (MUW) dataset” are further referred to as “combined dataset”.

Text data was parsed using python 2.7 and pandas 0.22. Plots and statistical analyses for NGS data were generated using R library maftools v.2.3.40 (39) and matplotlib 2.2.4 (40).

### Cell Culture

All *in vitro* experiments were performed on the human cell line derived from squamous cell carcinoma of the hypopharynx, namely FaDu (HTB-43; ATCC). Cells were typically cultured in 75 cm^2^ adherent cell flasks in DMEM D6429 medium (Sigma) supplemented with 10% HyClone calf serum (FBS, SH30072.03, GE Healthcare) and 1x antibiotic/antimycotic-solution (30-004-Cl, Corning) or penicillin-streptomycin solution (P4333, Sigma) during lentiviral infections. Cells were detached into suspension with 1x Trypsin (15090-046, Gibco), passaged in 2-3 day intervals and cryopreserved in liquid nitrogen in DMEM supplemented with 10% DMSO and 50% FBS. For routine passaging, cells were counted in trypan blue in Bürker chamber. For *in vitro* experiments, Count and Viability Kit for Muse Cell Analyser instrument (Luminex, USA) was used for more accurate counting. All cell cultures were monitored for Mycoplasma contamination by PCR weekly.

### Generation of CRISPR-Cas9 *KMT2C*-Mutant Cells and Clone Selection

CRISPR-Cas9 system was used to generate *KMT2C*-mutant (*KMT2C* ^mut^) FaDu cells. pLenti CRISPR v2 plasmid [Addgene #52961 (41)] was used along with lentiviral transfection to acquire stable expression of two different sgRNAs (sg2 and sg4), targeting exons 3 and 12 of *KMT2C*, respectively, as well as a non-targeting sgRNA control (sgNTC) (**Table 2**). *KMT2C*-targeting sgRNAs were designed using E-CRISPR (42) and CHOPCHOP tools (43); sg2 sequence was also previously included in the GeCKO v2 library (41). All used sgRNA sequences were predicted to affect the curated *KMT2C* transcript variant NM_170606.3 as well as most of putative transcripts except for XM_011516454.2, XM_017012489.1 and XM_017012490.2 lacking several exons at the N-terminus. HIV-SFFV-mRFP (received courtesy of dr Els Verhoeyen, Centre International de Recherche en Infectiologie, Lyon, France) plasmid was used as a transfection control. Lentiviral particles were assembled in HEK293 cells grown in DMEM supplemented with 10% FBS and PenStrep, using psPAX2 (Addgene #12260) and pMD2.G (Addgene #12259) plasmids. Virus-containing medium was acquired twice after subsequent overnight incubations and each time it was filtered with 0.45µm syringe filter, concentrated by overnight centrifugation, mixed in 1:1 proportion with fresh DMEM medium and added to FaDu cells. Then, 24h after second infection, puromycin selection was started at 2 µg/ml concentration previously measured to decrease viability of unmodified cells below 5%, with gradual decrease to 0.5 µg/ml over 1 week, resulting in a complete detachment of control HIV-SFFV-mRFP-expressing cells and acquisition of 100% or near 100% pLentiCRISPR transfected cell population as confirmed during further clonal selection.

**Table 2:** Sequences of sgRNAs used for KMT2C *in vitro* knockout in FaDu cell line.

sgRNA	Sequence	Targeted exon(NM_170606.3)	Affected transcripts
sg2	GACACAGATCGCTGAAGAGT	3	25/28
sg4	GCAGCTAATAAAGATGTCAA	12	26/28
sgNTC	ACGGAGGCTAAGCGTCGCAA	–	–

Induction of mutations by sgRNAs was confirmed by gel electrophoresis of PCR products amplified with primers flanking the sgRNA binding/Cas9 cut sites and subsequently using Sanger sequencing and NGS (**Supplementary Data 2.4** and data not shown).

FaDu cells expressing sg2 and sg4 were diluted to achieve an average concentration of 0.8 cells/per well when seeded on 96-well plates. Clones were detached from wells where single cells formed colonies and propagated through 24- and 6-well plates and 20 cm^2^ and 75 cm^2^ flasks when clone cell lines were frozen and DNA/RNA isolation was performed.

Clone pools and selected clones were compared to NTC-treated and non-treated cells using NGS to exclude the presence of significant off-target mutations and copy-number variations induced by CRISPR-Cas9.

All subsequent *in vitro* experiments were performed at least two times.

### Clonogenic Assay

Clonogenic assays for FaDu cell lines were carried out in standard 6-well plates with 625 cells per well. Cells were seeded in triplicates and cultured for approximately 14 days or until colonies started to merge, rinsed with PBS, and fixed and stained with methanol and 20% crystal violet solution.

After staining, plates were scanned with BioRad GS-800 scanner. Colonies were counted with ImageJ software with Fiji package (44) using *analyze particles* function (size: 0.0001–infinity, circularity 0.01–1.00), and area covered by colonies was measured for those automatically counted.

### DNA Synthesis Assay

For DNA synthesis measurements Click-iT EdU Imaging Kit (Thermo Fisher Scientific) was used and EdU incorporation was measured. Cells were seeded on 6-well plates in 0.15×10^6^ cells/well density in triplicates, incubated with 10 µM EdU for 1h the next day, detached with tripsin, fixed and stained with Alexa Fluor 488. Percentage of EdU-positive cells was measured on FACSCanto II cytometer (Becton Dickinson, USA). Exemplary gating is provided in **Supplementary Data 1.4**.

### Cisplatin-Sensitivity Assays

CellBlue and CellTiterGlo assays (Promega, USA) were used to measure cell sensitivity to cisplatin. Cells were seeded in 96-well plates in 5 replicates per each group in a density of 3×10^3^ cells per well and incubated for 24h prior to cisplatin addition. Cisplatin was added to achieve 2 µg/ml and 10 µg/ml final concentrations and the cells were treated for 48h. Viability measurements were taken as recommended by the manufacturer example 10 min after addition of CellTiter-Glo and 4h after addition of CellTiter-Blue. Fluorescence and luminescence were measured on Victor X4 instrument (Perkin Elmer), the latter in white opaque plates. Cell-free medium was used to measure background signal.

### Statistical Analysis of *In Vitro* Data

All *in vitro* data was analyzed using GraphPad Prism 6. For clonogenic and DNA synthesis assays, results for KMT2C-mutant cells were compared against NTC cells using Dunnett’s test. In cisplatin-sensitivity assays, average background-subtracted readouts from cisplatin-treated cells were recalculated as fractions of their corresponding controls and compared to NTC cells using Dunnett’s test.

## Results

### Integrated Analysis of Small-Scale Mutation and Copy-Number Variation Data From Laryngeal and Hypopharyngeal Cancers

To define the mutational profile in our cohort of patients (MUW dataset) comprised of laryngeal and hypopharyngeal cancers, we analyzed 37 genes previously described as significantly mutated in head and neck and esophageal carcinoma (14, 45–51) and covered by the probe designs used to enrich genomic libraries (**Supplementary Data 1.1–2**). Variants were manually curated to exclude benign and likely benign. Copy-number and loss-of-hetereozygosity (LOH) data was reanalyzed with the small-scale mutation data to investigate the interplay between these types of genetic aberrations.

**Figure 1:**
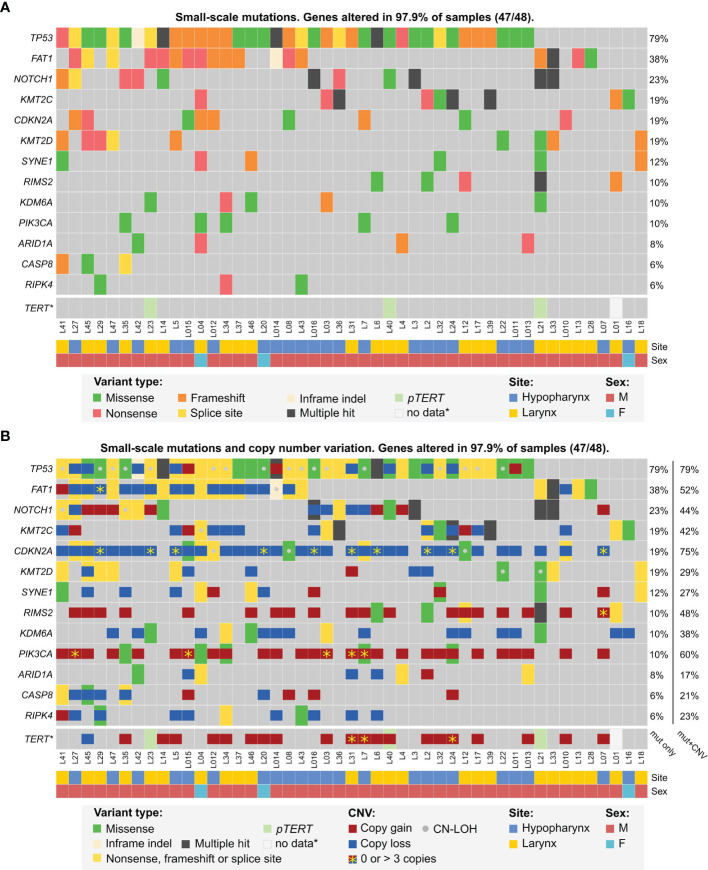
Mutational profile of laryngeal and hypopharyngeal cancer in the MUW dataset. Selected genes previously identified as significantly mutated were analyzed for small-scale mutations panel **(A, B)**, copy-number alterations and copy-neutral duplications (CN-LOH) panel **(B)**.

In this approach, 32 genes were altered by small-scale mutations in our dataset (**Supplementary Data 2.1, 3.1**) among which 13 genes were mutated in at least 3 patients (**Figure 1**). The top six mutated genes were *TP53*, *FAT1*, *NOTCH1*, *KMT2C*, *CDKN2A* and *KMT2D. TP53* was typically biallelically altered in most cases, either by multiple mutations or combinations of mutations and shallow deletions or copy-neutral losses-of-heterozygosity (CN-LOH), most frequently involving deletion of the short arm of chromosome 17. In some cases, putative amplification of mutated allele was found. *FAT1* was altered mostly by frameshift and nonsense mutations, accompanied by shallow deletions. *NOTCH1, KMT2C*, *CDKN2A* and *KMT2D* were similarly altered by truncating mutations and/or shallow deletions or CN-LOH. In particular, deep deletions of *CDKN2A* were detected in 20.8% of patients (10/48). Finally, 7 other genes were recurrently mutated in more than 2 patients (**Figure 1** and **Supplementary Data 2.1**).

Since *TERT* promoter was not covered by any of our sequencing panels, we used Sanger sequencing for this analysis. Mutations of *pTERT* were detected in 6.4% (3/47) patients, for which DNA was available. None of *pTERT*-mutated patients had any significant CNV of *TERT* gene, however, three other patients had *TERT* amplification (4–5 copies) (**Figure 1**).

A GISTIC analysis of CNV data from MUW dataset yielded a similar pattern of amplification and deletion peaks as in previously published TCGA HPVneg cohort (**Supplementary Data 2.2**). In the significantly amplified regions, multiple genes were found to have 4 or more copies recurrently, including *CCND1* (29%, 14/48 patients), *BIRC2/3* (8.3%, 4/48), *FGFR1* (10.4%, 5/48), *TP63* (14.6%, 7/48), *EGFR* (6.3%, 3/48), *ERBB2* (4.2%, 2/48), *MDM2* (4.2%, 2/48), *TERT* (6.3%, 3/48), *PIK3CA* (5/48), *MYC* (8.3%, 4/48), *MET* (4.2%, 2/48). *ERBB2*, *BIRC2/3*, *EGFR* and *CCND1* were highly amplified in some patients (in range of 39–52, 6–16, 5–15 and 4–11 copies, respectively). In the significantly deleted regions, *CDKN2A/B* (20.8% of patients, 10/48), *FAT1* (2.1%, 1/48) and *PTEN* (2.1%, 1/48) were affected by deep deletion.

A total of 18.8% of patients (9/48) harbored *PIK3CA* mutations and/or 4-5 gene copies and 6.3% (3/48) harbored *PTEN* mutation or deep deletion. In addition to the aforementioned copy number alterations, other well-known RAS pathway-activating events were detected in 8.3% patients (4/48): hotspot mutations in *HRAS* (1 patient), *PTPN11* (1) and *FGFR3* (1) and *NF1* homozygous deletion (1). Finally, one patient carried *KRAS* amplification (5 copies).

Genes implicated in DNA repair were affected in 18.8% of patients (9/48), either by mutation and deletion or CN-LOH (*BAP1*: 1 patient; *BRCA2*: 2; *CHEK2*: 1; *PMS2*: 1) or by mutation only (*ARID1A*: 4 patients, *BRCA1*: 1; *RECQL*: 1). Additionally, 25% of patients (12/48) were found to carry shallow deletions of *ATM* also identified as a significant GISTIC deletion peak yet without any associated *ATM* mutations.

Also, 4.2% (2/48) of patients carried potentially inactivating *CREBBP* mutations, similar to those previously described in leukemia (52). Truncating mutations in *ASXL1* in 8.3% (4/48) and *TET2* in 6.3% (3/48) patients were also detected.

**Figure 2:**
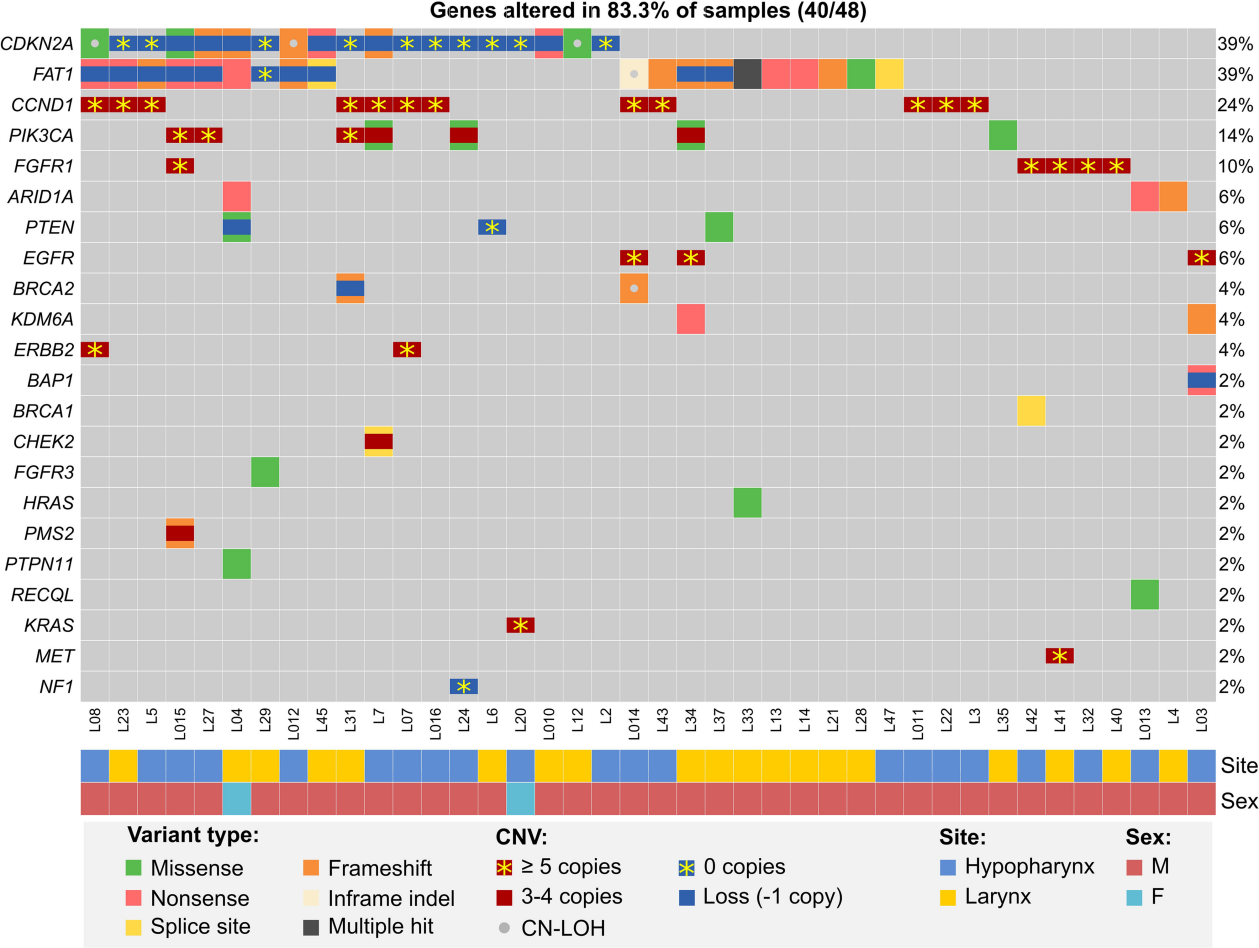
Potentially actionable or prognostic alterations in hypopharyngeal and laryngeal cancers from the MUW dataset. Included are pathogenic somatic mutation, amplifications (at least 3 additional copies), deep deletions and combinations of mutations and copy gains or possible second allele elimination due to copy loss or CN-LOH.

We then analyzed NGS data for potentially druggable or prognostic genetic alterations. We selected only previously described mutations or those with strong prediction of pathogenicity, deep deletions and amplifications to at least 5 copies, while eliminating shallow deletions without a second hit, low-level amplifications and other ambiguous alterations. With this strict approach, we found that over three-quarters of patients harbored at least single alterations of genes involved in RTK/RAS/PI3K, cell-cycle or DNA damage repair pathways (**Figure 2** and **Supplementary Data S3.3**).

While we could not reliably assess tumor mutational burden due to limited availability of paired germline samples, we analyzed copy numbers of *CD274/PDCD1LG2* and found that 29.2% (14/48) patients had shallow deletions and 12.5% (6/48) had copy gains (3–4 copies) of these genes, potentially affecting PD-L1 and PD-L2 expression (53) and sensitivity to immune checkpoint inhibitors.

### Mutational Profile of Hypopharyngeal Cancer

**Figure 3:**
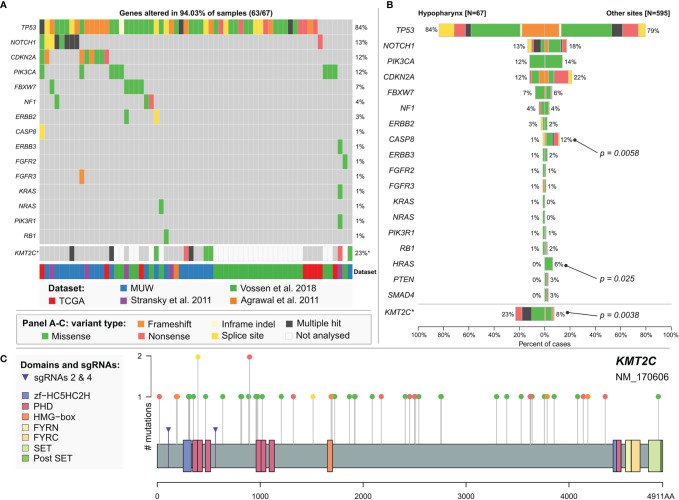
**(A)** Mutational profile of hypopharyngeal cancers. Chart based on a combined dataset and selected genes common to all datasets. **(B)** Comparison of mutation frequency in selected genes between hypopharyngeal and non-hypopharyngeal head and neck cancers. *KMT2C mutation frequency is calculated separately excluding NKI dataset (Vossen et al.) in which this gene has not been sequenced. Significance is calculated using maftools mafCompare function (Fisher’s exact test). **(C)** *KMT2C* (MLL3) mutations in head and neck cancers. Additional markers (purple arrows) indicate positions of mutations induced by CRISPR sgRNAs 2 and 4 in FaDu *KMT2C* ^mut^ cell lines. Chart is based on a combined dataset except for NKI data.

Finally, to better determine the mutational profile of hypopharyngeal cancers we used data from the MUW dataset as well as previously published data for hypopharyngeal cancer samples with confirmed negative HPV infection status (combined dataset, **Supplementary Data 3.6**). In this analysis, we included 18 significantly mutated genes common to all datasets (listed in **Supplementary Data 1.1**). To minimize the differences in variant filtering between datasets we added 6 variants detected in the MUW cohort that we excluded from previous analyses due to low likelihood of pathogenicity. In total, 595 non-hypopharyngeal and 67 hypopharyngeal cancers were compared using *maftools*. We did not find any significant differences (**Figures 3A, B** and **Supplementary Data 2.3**) in mutation frequency except for *CASP8* mutations, which were very rare (*p* = 0.0058, OR 0.12, CI 0.0028–0.69) and *HRAS* mutation, which were absent (*p* = 0.025, OR = 0, CI 0–0.87) in hypopharyngeal cancers.

### *KMT2C* (MLL3) Mutations in Head and Neck Cancer

In our dataset, we identified mutations in *KMT2C* gene, encoding Histone-Lysine N-Methyltransferase 2C, in 14 (29%) or 9 (18.8%) patients, after elimination of benign variants. Seven (14.6%) patients carried *KMT2C* truncating mutations and we also observed frequent shallow deletions or combinations of mutation and CNV/CN-LOH (**Figure 1**). In the combined HPVneg dataset (excluding NKI dataset, Vossen et al., 2018) *KMT2C* mutations were present in 8.7% (48/551) of patients (**Supplementary Data 2.3, 3.7**) and 3.4% (19/551) of them harbored truncating mutations that were scattered across the gene (**Figure 3C**). Moreover, we found that hypopharyngeal cancers harbored more *KMT2C* mutations than cancers from other sites (**Figures 3A, B** and **Supplementary Data 2.3**), but this difference is likely not biologically relevant as discussed below.

*KMT2C* was already characterized as a tumor suppressor gene in acute myeloid leukemia (54). Based on above-mentioned data and our findings in laryngeal and hypopharyngeal cancer, we decided to assess the biological effects of *KMT2C* mutation in commercially available HPV-negative hypopharyngeal cell line FaDu (ATCC HTB-43). First, we characterized FaDu cells using targeted NGS and found multiple pathogenic genetic aberrations, including mutations of *TP53* (missense and splice-site), *CDKN2A* (splice-site, homozygous), *FAT1* (frameshift, homozygous), *SYNE1* (missense, frameshift), deep deletions of *AJUBA* and *SMAD4*, high-level amplification of *CCND1*, as well as mutations in *ERBB3*, *VHL* and other genes (**Supplementary Data 3.4**). Hence, we found that FaDu harbors multiple genetic lesions frequently occurring in head and neck and esophageal cancer.

### Induction of *KMT2C* Mutations by CRISPR-Cas9

To study the role of truncating mutations in *KMT2C* in HNSCC, we used CRISPR-Cas9 to induce mutations in the N-terminal quarter of the coding sequence (**Figure 3C**). We generated *KMT2C* ^mut^ FaDu cells stably expressing two different *KMT2C*-targeting sgRNAs (sg2 and sg4) as well as a non-targeting control sgRNA (sgNTC). Using PCR, Sanger sequencing (data not shown) and NGS (**Supplementary Data 2.4**) we confirmed that both sgRNAs effectively induced indels while sgNTC did not, additionally revealing that most clones derived from pools harbored more than 2 copies of *KMT2C*, in concordance with the expected hyperdiploidy of FaDu cells and ploidy analysis in selected cell populations (data not shown). Based on initial growth observations we selected clones sg2-7 and sg4-14 along with clone pools sg2 and sg4 for further experiments.

Targeted NGS analysis of CRISPR-Cas9 modified cells confirmed the specific induction of various *KMT2C* mutations at exons 3 and 12 (**Supplementary Data 2.4**). Furthermore, through Mutect-2 comparisons and manual analysis of NGS data, we did not find any additional, significant genetic point mutations or CNVs unique to any of the modified cell pools, except for clone sg4-14 which could be distinguished by lack of several variants (**Supplementary Data 3.4**) as well as by more pronounced deletion of 3p11.1/*EPHA3*.

### The *In Vitro* Effects of *KMT2C* Mutations on Proliferation and Cisplatin Resistance of Hypopharyngeal Cell Line FaDu

**Figure 4:**
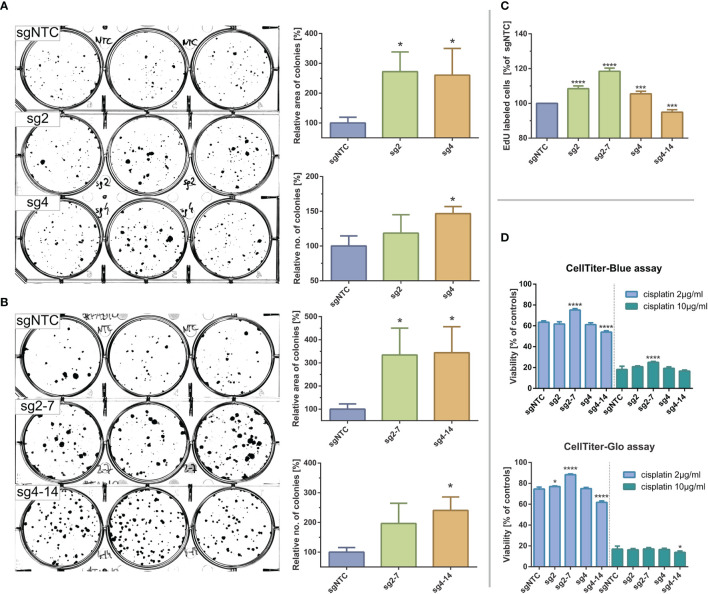
Effects of CRISPR-Cas9 mediated *KMT2C* mutations on clonogenic potential and cisplating sensitivity of FaDu hypopharyngeal cancer cell line. **(A, B)** Clonogenic assays for clone pools **(A)** and clones **(B)**. Significant increases in colony size and number can be observed in *KMT2C* ^mut^ cells as compared to control. **(C)** DNA synthesis/EdU incorporation assay. EdU incorporation is significantly increased in *KMT2C* ^mut^ cells, except for sg4-14 clone. **(D)** Cisplatin sensitivity measured with CellBlue and CellGlo viability assays. Results do not indicate a clear association between *KMT2C* loss and cisplating sensitivity. Control cells (sgNTC) are transduced with non-targeting sgRNA. Asterisks indicate statistical significance, *p-value* in Dunett’s test * 0.05/*** 0.001/**** 0.0001. Error bars represent standard deviation.

We checked whether induction of *KMT2C* mutations affects phenotypic features of FaDu cells and, therefore, firstly assessed the clonogenic potential of *KMT2C* ^mut^ FaDu cells by the clonogenic assay on 6-well plates. We noticed a marked increase in clonogenicity of the modified cells as compared to sgNTC-expressing cells, as manifested not only by significant differences in number of colonies but also in the growth surface area. These effects were confirmed in sg2 and sg4 clone pools (1.19x and 1,47x more colonies and 2.72x and 2.61x larger growth area, respectively) and clones sg2-7 and sg4-14 (1.97x and 2.41x more colonies and 3.34x and 3.44x larger growth area, respectively) (**Figures 4A, B**).

We could not reliably analyze the cell cycle using propidium iodide due to variable ploidy among clone pools and clones (data not shown). Instead, we analyzed DNA synthesis rate using EdU incorporation assay. Significantly increased uptake was observed for *KMT2C* ^mut^ cells (8.5%, 5.5% and 18.5% increase for sg2, sg4 and sg2-7, respectively), except for sg4-14 clone (5.1% decrease), confirming their proliferative advantage over sgNTC cells (**Figure 4C**).

Finally, to test if *KMT2C* mutation could affect response to standard chemotherapy, we measured viability of FaDu cells exposed for 2 days to two different cisplatin concentrations using CellTiter-Blue and CellTiter-Glo (Promega) assays. While some statistically significant differences in cisplatin sensitivity were observed for clones sg2-7 and sg4-14, they could not be clearly associated with *KMT2C* mutation, given their magnitude and distribution (**Figure 4D**).

## Discussion

In this work we described the molecular landscape of HPV-negative head and neck tumors located in the hypopharyngeal and laryngeal regions. In our dataset, we detected molecular alterations, which were previously described in high-throughput head and neck cancer studies (13–15, 49, 50), as well as new ones. Many of these genetic alterations have a well-established or at least a putative role in molecular oncogenesis. Importantly, in the majority of the analyzed tumors, we found potentially targetable vulnerabilities or prognostic markers.

As expected, the most frequently mutated gene was *TP53*, altered in almost 80% of our samples. Mutations in *TP53* are found in the majority of HPVneg head and neck tumors and were shown to affect survival, also in hypopharyngeal cancer; specifically, truncating mutations (frameshift, nonsense, splice-site) seem to be associated with poorer outcome (55, 56). In our study, 45.8% (22/48) patients from the MUW dataset and 43.3% (29/67) of the hypopharyngeal cancer patients in the combined dataset carried truncating *TP53* mutations, possibly leaving others with a more favorable prognosis.

The frequent alterations of cell-cycle genes *CDKN2A* and *CCND1* could predict sensitivity to CDK4/6 inhibition, even if predictive value has not been definitely confirmed yet (57). Conversely, *FAT1* and *RB1* loss has been shown to negatively affect efficacy of CDK4/6 inhibitors (58). While 50% (24/48) of our patients harbored mutations in *CDKN2A* and/or *CCND1* (**Figure 2**), 45.8% (11/24) of them carried concurrent and mostly biallelic *FAT1* alterations that could result in primary resistance. Given the promising clinical activity of palbociclib in HPVneg HNSCC (59), molecular testing for those genes could identify patients with the greatest likelihood of response. *FAT1* mutations were also recently associated with progression of HNSCC, which is particularly important given the high mutation rate demonstrated here and in previous studies (49, 60).

Genes belonging to RTK/RAS/PI3K pathways were affected by evident pathogenic alterations in 43.8% (21/48) of patients (**Figure 2**) – most of these were RTK amplifications and infrequent hotspot mutations, possibly sensitizing tumors to a wide variety of single-drug or combination therapies. Among other notable genes, *KDM6A* truncating mutations were observed only in two patients (in one of which the mutation was clearly subclonal). Other alterations in this gene included missense mutations and possible deletions on chromosome X. Since *KDM6A* loss has been shown to sensitize cancer cells to EZH2 inhibition (for example to FDA-approved tazemetostat) (61) and most of our patients were men, further studies would be desirable. Three patients were found to have truncating *ARID1A* mutations. *ARID1A* mutations negatively impact DNA damage repair in cancer cells and while their role in regulating sensitivity to checkpoint inhibitors is unclear (62, 63), they have been also shown to sensitize cancer cells to PARP inhibition (64, 65) as well as to EZH2 inhibition (66). Other genes implicated in the DNA damage sensing and repair family were also mutated in our cohort in 16% of patients, resulting in an opportunity to use synthetic lethality approach.

We and other authors found frequent Notch pathway mutations. *NOTCH1* and Notch pathway are generally regarded as having a tumor suppressor function in head and neck cancer (67) which hampers the possibility to use γ-secretase inhibitors that are in development for cancers with Notch pathway activation (68). However, *NOTCH1* mutations (69) together with *FAT1* (70) and *AJUBA* mutations (71, 72), which all occur in head and neck cancers, could possibly converge on WNT/β-catenin pathway activation. Since multiple inhibitors of this pathway are in development (73), these mutations can become actionable in future.

Using previously published and new data, we further analyzed the mutational profile of hypopharyngeal cancer and showed that despite poorer prognosis, it does not differ significantly from other head and neck cancers in terms of mutation frequency in major genes (**Figure 3B**) which remains in agreement with previous reports (15). Further analyses on larger cohorts and including CNV data are therefore still required to identify any differences between tumors located in different subsites. We confirmed previous observations that laryngeal and pharyngeal cancers rarely harbor *CASP8* and *HRAS* mutations in comparison to oral cancers (15) – in our comparison, these alterations were almost completely absent in hypopharyngeal tumors when juxtaposed with other head and neck cancer subtypes. Moreover, according to a curated set of non-redundant studies in cBioPortal*, CASP8* and *HRAS* mutations, they seem to be generally more frequent in head and neck cancers (9.57% and 5.83%, respectively) than in gastroesophageal adenocarcinomas and squamous cell carcinomas (2.07% and 0.15%).

We have detected *pTERT* mutations in 6.4% (3/47) of patients, all originating from larynx (**Figure 1**). Recent reports indicate that *pTERT* mutations are typically found in oral cancers (up to more than 50%) and rare or absent in other sites (74–76), therefore our results again reaffirm these findings. Consequently, *pTERT* mutations also seem to be very rare in esophageal squamous cell carcinoma (77).

Expression changes and mutations in genes encoding histone methyltransferases have been widely recognized in squamous cell carcinomas (78) and in cancer in general (79). While the exact nature and roles of these aberrations in specific cancer types are disputable, extensive experimental evidence suggests that histone methyltransferases frequently serve as tumor suppressors. In our cohort, we found *KMT2C*, *KMT2D* and *NSD1* to be frequently affected by small-scale mutations and copy number alterations (**Figure 1**, and **Supplementary Data S2.1**). *NSD1-* or histone H3-mutated tumors have been found to constitute a hypomethylated subset within HPVneg HNSCC (80) while *NSD1* and *NSD2* mutations have been associated with favorable prognosis in laryngeal cancers (81). *KMT2D* mutations have been recently found to sensitize cancer cells to aurora kinase inhibitors in HNSCC (82). Data on the *KMT2C* role in HNSCC is very limited and relatively frequent mutations in our cohort prompted us to conduct additional analyses.

Chen et al. identified *KMT2C* as a target of deleterious mutations as well as copy losses at chromosome 7 and showed its tumor-suppressive role in acute myeloid leukemia, though KMT2C knockdown was incapable to drive oncogenesis alone (54). In the context of frequent truncating and missense *KMT2C* mutations in breast cancer, Gala et al. found *KMT2C* loss to have both tumor-promoting and tumor-suppressive role depending on the estrogen availability (83). Cho et al. described frequent *KMT2C* missense mutations in diffuse-type gastric adenocarcinoma that translated into diminished protein expression, which then could be associated with worse prognosis, but only in diffuse-type adenocarcinoma. In the same study, *KMT2C* loss also induced epithelial-to-mesenchymal transition, including enhanced migration and invasion capabilities (84). Rampias et al. observed that loss of *KMT2C* activity in bladder cancer and others does not directly affect proliferation or viability but causes DNA repair defects and sensitizes cells to PARP inhibition by downregulation of genes involved in homologous repair of double-strand breaks (85). This data collectively shows pleiotropic and context-dependent functions of KMT2C and consequences of its aberrations.

KMT2-family genes are altered by various types of mutations in multiple positions. Specifically, *KMT2C* mutations can be located in the SET domain or the PHD domain clusters but are also found in the rest of the gene body and this applies both to missense and truncating variants (79). As a result, different classes of mutations likely have distinct biological effects, for example, it has been shown that the loss of catalytic activity of *KM2TC* has largely distinct effects from those observed for complete gene inactivation (86, 87). We identified both missense and truncating mutations in *KMT2C*, yet missense mutations were mostly predicted to be benign or of unknown significance. Moreover, only 6/35 missense *KMT2C* mutations from the combined HNSCC dataset were located in previously described hotspot regions in *KMT2C* (88), being rather scattered across the gene instead.

Some tumors in the MUW dataset carried *KMT2C* mutations with low (<10%) VAFs. Tumors L39 and L36 had concurrent mutations in *TP53* with similar, low VAFs while tumor L01 carried mutations in *KMT2C, FBXW7, RIMS2* and *NF1* with VAF in a range of 7-17%; in these cases, *KMT2C* mutations were probably clonal and their VAF reflected lower tumor content in the sample. Tumors L2, L32, and L24 carried concurrent *TP53* mutations with much higher VAFs – in these cases, *KMT2C* mutations were likely subclonal. Tumor L16 did not have any additional mutations which suggests low tumor content or distinct genetic background.

We used CRISPR-Cas9 to induce *KMT2C* inactivation, which should mimic the effects of randomly dispersed truncating mutations described here and by others. This allowed us to find that in cell line FaDu loss of *KMT2C* provides a proliferative advantage, supporting its tumor-suppressive role in hypopharyngeal cancer. Importantly, our results are supported by previously published data on esophageal cancer cell lines (89). It should be emphasized, though, that the effects we measured are possibly limited only to a fraction of cells (**Figures 4A, B**), which may imply that *KMT2C* disruption is a cooperating event rather than a strong driver of oncogenesis and its outcomes originate from an interplay of many unidentified factors. It is worth mentioning in this context, that in our dataset *KMT2C* mutations co-occurred with *TP53* mutations in most cases. As reviewed by Fagan and Dingwall, *KMT2D/KMT2C/TP53* genes were shown to cooperate and their mutations were found to be simultaneously present in various cancers, potentially affecting survival. These authors also suggested that significant co-occurrence of *KMT2C/KMT2D* mutations with *TP53* (and several other) mutations may indicate their role in epigenetic priming of early tumor cells for acquisition of additional genetic mutations (79).

We did not obtain a conclusive data linking *KMT2C* mutation to cisplatin sensitivity (**Figure 4D**) which could support previous data on DNA damage repair deficiency (85). Finally, we found *KMT2C* to be more frequently mutated in cancers originating from the hypopharynx than from other sites. However, this difference likely resulted from the limited cohort size as well as inclusion of possibly benign, low-VAF variants in the calculation, that were identified in our samples.

Our study has limitations that warrant further research. We have found a limited number of differences in mutation spectrum between cancers located in the hypopharynx and others sites, such as larynx, even when combined with previously published data. Additionally, our dataset for hypopharyngeal cancers is limited in respect to cohort size and analyzed genetic alterations. Our data also may not comprise all mutations because of technical reasons and lack of germline tissue. Normal DNA samples were available only for 13/48 patients, making it more complicated to discriminate between somatic mutations and rare germline variants. Finally, due to scarcity of reliable hypopharyngeal cancer cell lines, our patient-based data is supported by experiments involving an applicable cell line, namely FaDu, though it should be noted that this cell line has a genetic profile typical for this type of cancer.

In summary, we provide a new insight into the molecular landscape of hypopharyngeal cancer and identify potential new biomarkers. Importantly, our results suggest a tumor-suppressive role of *KMT2C* in hypopharyngeal cancer, similarly as in other solid tumors and hematological malignancies.

## Data Availability Statement

The original contributions presented in the study are publicly available. This data can be found in the **Supplementary Material** as well as the European Nucleotide Archive, accession no: PRJEB50065.

## Ethics Statement

The studies involving human participants were reviewed and approved by Bioethics Committee of Medical University of Warsaw. The patients/participants provided their written informed consent to participate in this study.

## Author Contributions

Conceptualization, MMM, EO-W, and TS; methodology, MMM, JIJ, and TS; software, MMM and PS; formal analysis, MMM; investigation, MMM, AR, JIJ, MP, KP, AK, MR, JG, and MW; resources, AR, BG, and EO-W; data curation, MMM, AR, PS, JG, and TS; writing—original draft preparation, MMM, AR, and TS; writing—review and editing, JIJ, MP, and TS; visualization, MMM; supervision, BG, RP, EO-W, and TS; funding acquisition, MMM and TS. All authors have read and agreed to the published version of the manuscript.

## Funding

This research was funded by the Polish National Science Centre, grant number 2013/09/N/NZ2/01354. MM and MP were supported by the Postgraduate School of Molecular Medicine, Medical University of Warsaw.

## Conflict of Interest

The authors declare that the research was conducted in the absence of any commercial or financial relationships that could be construed as a potential conflict of interest.

## Publisher’s Note

All claims expressed in this article are solely those of the authors and do not necessarily represent those of their affiliated organizations, or those of the publisher, the editors and the reviewers. Any product that may be evaluated in this article, or claim that may be made by its manufacturer, is not guaranteed or endorsed by the publisher.
